# Structural and evolutionary insights into understudied bacterial serine–threonine pseudokinase families

**DOI:** 10.1042/BST20253080

**Published:** 2025-09-11

**Authors:** Brady O’Boyle, Debarshi Ryan Bhowmik, Patrick A. Eyers, Dominic P. Byrne, Natarajan Kannan

**Affiliations:** 1 Department of Biochemistry and Molecular Biology, University of Georgia, Athens, GA, 30602, U.S.A.; 2 Institute of Bioinformatics University of Georgia, Athens, GA, 30602, U.S.A.; 3 Department of Biochemistry, Cell and Systems Biology, Institute of Systems, Molecular and Integrative Biology, University of Liverpool, Liverpool, L69 7ZB, U.K

**Keywords:** bacterial kinases, evolutionary biology, protein structure, protein–serine–threonine kinases, pseudokinases

## Abstract

Pseudokinases, once considered catalytically inactive remnants of evolution, have emerged as key regulators of numerous fundamental biological processes. While eukaryotic pseudokinases have attracted significant attention, bacterial pseudokinases remain largely unexplored experimentally. Recent advances in sequence analysis and structural modeling have identified and characterized multiple conserved bacterial pseudokinase families, each with distinct predicted catalytic impairments but unknown functions. This review delves into their classification, structural features, and evolutionary adaptation. We also highlight the significance of bacterial pseudokinases in host–microbe interactions and their emerging potential as therapeutic targets. By integrating bioinformatics with experimental approaches, future research is poised to uncover the biological functions of bacterial pseudokinases, providing new insights into microbial signaling mechanisms and revealing new strategies to interrogate bacterial cell signaling, including pseudokinase drivers of infection and antimicrobial drug resistance.

## Introduction

Protein phosphorylation is one of the most common forms of post-translational modifications (PTMs) across all domains of life. In eukaryotes, amino acid phosphorylation is catalyzed by a large family of evolutionarily related protein kinases broadly classified as serine/threonine (S/T) or tyrosine kinases based on the residues they phosphorylate in protein and peptide substrates. Phosphorylation by protein kinases regulates diverse cellular processes [1–3], and abnormal protein kinase activity is causally or directly associated as a driver for many human diseases. Within eukaryotes, about 10% of all serine–threonine protein kinases (STKs) lack one or more of three catalytic residues considered necessary for ATP-dependent phosphorylation: the ATP-binding β3 lysine, the HRD-Asp catalytic base, and/or the metal-binding DFG-Asp [2–5]. Although variations of this common theme naturally exist within large superfamilies such as kinases, mutations of any of these three residues in a kinase sequence provide appropriate evidence for these proteins to be considered pseudokinases, especially in the absence of experimental data demonstrating catalytic activity [6,7]. Indeed, various lines of experimental evidence demonstrate that pseudokinases are generally, but not always, catalytically inactive and/or deficient in nucleotide binding [8]. Pseudokinases were originally thought to be non-functional remnants of obscure, or even dead-end, evolutionary processes but have since been shown to perform a myriad of rate-limiting non-catalytic (or atypical catalytic) functions. Moreover, pseudokinases form part of a much broader set of ‘pseudoenzymes,’ which have evolved for an array of cellular functions across life even in the absence of classical catalytic outputs [3]. These include allosteric activation of partner proteins, which are themselves often canonical kinases [2,3,9–16], switching-based signaling [2,3,17–19], competitive inhibition through sequestration within the pseudokinase domain [2,3,20], and/or regulated scaffolding roles [2,3,21–24], some of which are druggable allosterically [25,26]. Furthermore, examination of pseudokinases, notably bacterial and human proteins with enzymatic activities that diverge from the classical metal-dependent phosphotransferase reaction [27–29], continues to illuminate the expanding biological functions of canonical kinases, for which kinase-independent roles are now widely established throughout life [2,28,30].

## Bacterial serine–threonine kinases are distinct from other bacterial protein kinases

**Figure 1:**
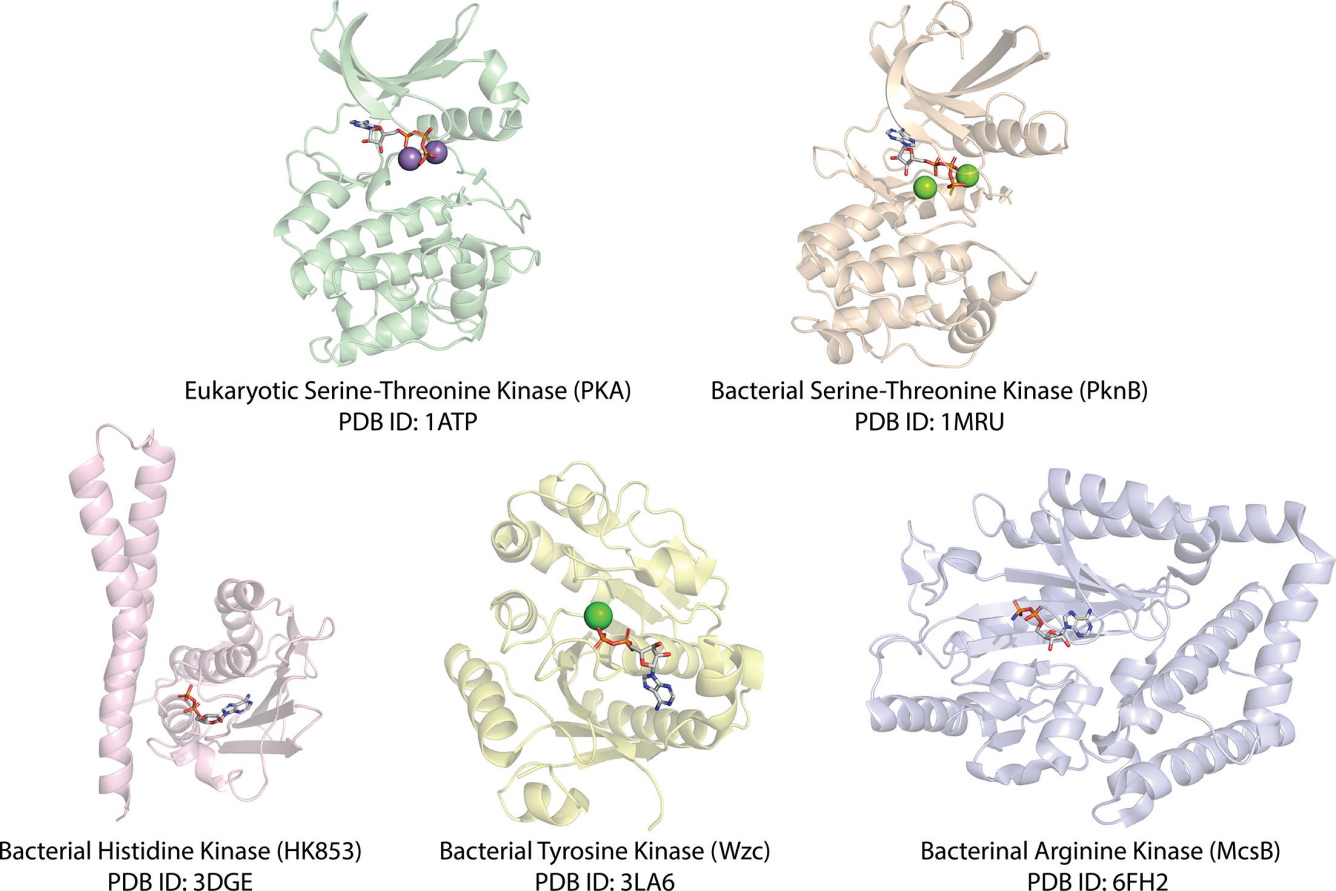
Structural comparisons between eukaryotic STKs and bacterial kinases. Crystal structures of each kinase family are shown bound to a nucleotide, with magnesium (green sphere) or manganese (purple sphere).

Bacterial genomes encode numerous biochemically defined protein kinase activities [31]. Historically, bacterial phosphosignaling has been predominantly associated with histidine kinases, responsible for histidine autophosphorylation and aspartate phosphotransfer as part of bacterial ‘two-component systems’ [32,33]. However, protein kinase enzymes with specificity toward tyrosine, arginine, and S/T side chains in prokaryotic systems have also been identified [34–36]. These four prokaryotic kinase families—termed histidine, arginine, tyrosine, and serine–threonine kinases—are key regulators of a myriad of biological processes, ranging from cell development [37,38], macromolecule synthesis [39,40], protein quality control [34,41,42], signal transduction [32], and stress responses [43–45] to virulence [39,40,46–50], infection [51] and antibiotic resistance [40,40,46,52,53], demonstrating the complexity and necessity of phosphorylation-based signaling within bacteria. Despite all facilitating ATP-dependent phosphorylation, each of these families is evolutionarily and structurally distinct from each other (Figure 1). Only bacterial serine–threonine protein kinases [here termed bacterial STKs (bSTKs)] share structural homology with eukaryotic serine–threonine (and eukaryotic tyrosine) kinases. In contrast, bacterial histidine, arginine, and tyrosine kinases lack the characteristic bi-lobal structures that define the eukaryotic protein kinase fold, which creates the familiar protein kinase ATP-binding pocket. As these are evolutionarily distinct from bSTKs, they will not be discussed further here.

bSTKs share a clear common ancestry with eukaryotic canonical kinases and pseudokinases [54]. Importantly, bacterial pseudokinases are also known to exist, although their prevalence in individual kinomes is more variable than the ‘10%’ rule of thumb [55,56] established for most eukaryotes. Indeed, based on the simple active site definitions, some bacterial kinomes possess no pseudokinase-like sequences (i.e. they possess all three canonical ATP-binding and phosphotransfer residues in all identified STKs), whereas others encode as many as 67% pseudokinase sequences [55]. Our current understanding of bacterial pseudokinases is still somewhat limited when compared with their eukaryotic counterparts. As a result, we possess minimal detailed molecular information on the biological functions of these ‘degraded’ kinases, many of which probably operate in specialized biological niches that have emerged from the structurally conserved stable protein kinase fold. Despite our current rudimentary understanding, studies of individual bacterial pseudokinases are beginning to reveal some of their secrets, with examples of diverse and essential biological roles emerging in various prokaryotic species.

**Figure 2:**
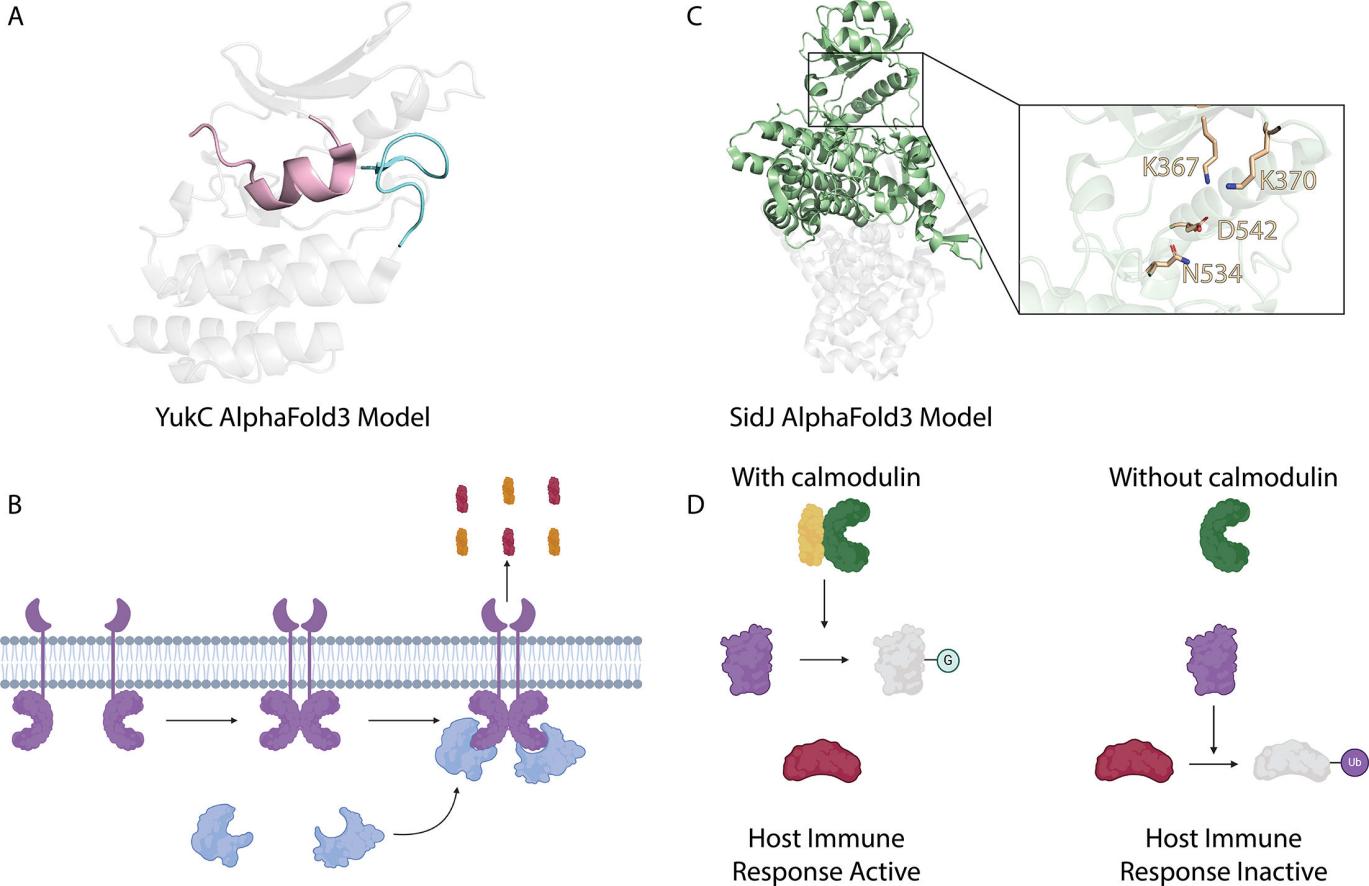
Examples of the functional diversity of bacterial pseudokinases. (**A**) Crystal structure of YukC of *Bacillus subtilis* (PDB ID: 6Z0F). The degraded glycine-rich loop is shown in pink, and the markedly reduced activation loop is shown in blue. (**B**) The mechanism and function of YukC of *B. subtilis*. YukC (purple) is a monotopic transmembrane protein, which recruits the machinery of the secretion apparatus (blue) upon dimerization. The complex then secretes effector proteins (orange and red) to lyse host phagosomes and promote pathogen survival. (**C**) Crystal structure of SidJ full-length protein and the canonical active site of the kinase domain (PDB ID: 6OQQ). The kinase domain is shown in green, and key residues within the active site required for glutamylase activity are shown in light brown. (**D**) The function of SidJ of *Legionella pneumophila* in the presence and absence of host-derived calmodulin. In the presence of calmodulin (yellow), SidJ (green) functions as a glutamylase for SdeA (purple). Glutamylation inactivates SdeA and prevents it from ubiquitinating host Rab GTPases involved in protein trafficking (red). Without calmodulin, SidJ is inactive, and SdeA can ubiquitinate multiple host Rab GTPases, dampening the host immune response.

One such example is EssB of *Staphylococcus aureus* (also known as YukC in *Bacillus subtilis*), a transmembrane pseudokinase found in Firmicutes. The pseudokinase domain exhibits degradation in all three catalytic motifs, as well as a degraded glycine-rich loop (which normally accommodates the adenine moiety of ATP), and an attenuated activation loop (which normally enables ATP and substrate binding upon phosphorylation) (Figure 2A). Despite this, EssB/YukC plays a critical structural and regulatory function in the Type VII secretion system b (T7SSb) [23,57–61]. This system is essential for the secretion of survival-promoting effector proteins, such as LXG toxins or WXG100 proteins, that induce host phagosome lysis and serve as toxins in antibacterial competition [23,58,59,62–64]. Mechanistically, the cytoplasmic pseudokinase domain of EssB/YukC plays a non-catalytic role in the assembly and secretion of effector proteins, primarily functioning in protein scaffolding and recruitment, as well as stabilizing EssB/YukC homodimerization (Figure 2B) [23,59–61]. These non-catalytic functions highlight the fundamental significance of pseudokinases in bacterial physiology, demonstrating how they can serve as structural hubs that co-ordinate complex cellular processes. More broadly, the role of EssB/YukC underscores the evolutionary adaptability of pseudokinases, which, despite lacking enzymatic activity, remain essential for regulatory and structural functions in diverse biological systems.

While EssB/YukC exemplifies the regulatory scaffolding functions of some pseudokinases, other bacterial pseudokinases have evolved entirely new enzymatic activities [27,65]. One such example is SidJ, an effector protein of the *Legionella pneumophila*, an opportunistic bacterial pathogen of humans and the causative agent of Legionnaires’ disease (Table 1). SidJ catalyzes ATP-dependent polyglutamylation of the catalytic glutamate residues of the mono-ADP-ribosyl transferase domain of SdeA, a member of the SidE family of ubiquitin system disrupting effectors [66–68]. SidJ does not retain a canonical catalytic motif; instead, several residues within the active site are required for glutamylase activity, including the repurposed canonical ATP- and metal-binding residues (K367 and D542) [65,66,68–70] (Figure 2C). Glutamylation of SdeA by SidJ abrogates phosphoribosyl ubiquitination of multiple host Rab GTPases, suppressing host immune responses and fine-tuning immune evasion to balance bacterial survival while maintaining host cell integrity [66,67] (Figure 2D). Regulation of SdeA in this manner prevents excessive host cell damage and establishes cellular conditions more conducive to bacterial replication [66,69]. Another pseudokinase that exhibits new enzymatic functions is SdjA, a SidJ paralog in *L. pneumophila* that exhibits both glutamylation and deglutamylation activity [70,71]. Glutamylation, however, is not the only example of enzymatic divergence among bacterial pseudokinases. SelO is a conserved pseudokinase family found across eukaryotes and bacteria, including *Escherichia coli* and *Pseudomonas syringae* [27]. These pseudokinases have been shown to facilitate AMPylation, in which adenine monophosphate (AMP) is derived from ATP and transferred to a substrate to regulate cellular processes such as redox homeostasis [27]. The potentially unique enzymatic activity of SidJ, SdjA, and SelO highlights the evolutionary plasticity of pseudokinases, demonstrating their ability to acquire alternative catalytic functions beyond traditional ATP-dependent phosphorylation.

**Table 1:** Pseudokinases detected in pathogenic bacteria.

Bacterial species	UniProt ID	Gene name	Pseudokinase domain	bSTK family	ATP-binding motif (VAIK)	Catalytic motif (HRDxxxxN)	Metal-binding motif (DFG)
*Mycobacterium leprae*	Q9CCX9	-	728–960	ActPs3	VALT	Lsidhpsr	Y--
*Mycobacterium tuberculosis*	P9WJK3	mviN	710–942	ActPs3	VALT	Lsidhpsr	Y--
*Nocardia asteroides*	U5ED14	-	862–1102	ActPs3	VALT	Lsidhpdr	FPG
*Pseudomonas aeruginosa*	Q9I0B5	-	113–360	DLHK	AVLT	Hrdlrpas	GFG
*Leptospira interrogans*	Q8F6Y5	-	9–269	CP1	KILR	Hnqigpss	WLG
*Leptospira interrogans*	Q8F1M0	-	6–268	CP1	VLIK	Hkslcpgn	DFS
*Yersinia pestis*	A0A380PMX1	-	19–348	Yegi1	VVA-	Hsdlsykn	DVD
*Nocardia asteroides*	U5EIF8	-	17–269	Act1	VVVR	Hqrlrads	EIG
*Legionella pneumophila*	Q5ZTK6	sidJ	347–739	Unclassified	VAVK	Fpqladifh	DLG
*Staphylococcus aureus*	P0C053	essB	9–211	Unclassified	LDMR	Ytfvlapd	TRG
*Escherichia coli*	P77649	SelO	67–333	Unclassified	WHLK	Hgvmntdn	DYG
*Pseudomonas syringae*	Q87VB1	ydiU	73–339	Unclassified	LHLK	Hgvmtdn	DFG

The remarkable functional diversity of bacterial pseudokinases, exemplified by EssB/YukC and SidJ, raises several key questions: how many other bacterial pseudokinases exist? What functions do they perform? And how do their unique structural features enable those functions? Large-scale evolutionary analyses offer valuable insight into the sequence and structure-function diversity of these proteins by identifying conserved pseudokinase families and the structural constraints that shape their functional adaptations.

A recent study classified >300,000 bSTK protein sequences into 42 conserved families based on evolutionarily conserved residue patterns [72], known as constraints [73–75]. These constraint patterns are believed to be critical in defining family-specific functions and regulatory mechanisms. Among these 42 families, five—ActPs1, ActPs2, ActPs3, DLHK, and glycine-leucine-tryptophan (GLW)—were composed entirely of pseudokinase sequences, while two others, CP1 and aspartate–tyrosine–aspartate (DYD), contained a high proportion of pseudokinases. These families exhibit significant diversity in their catalytic motifs, structural features, and associated non-kinase domains, highlighting their broad functional potential.

For clarity, these seven families will be categorized into two groups based on the degree of conservation or divergence in their catalytic motifs: (i) families with divergence in all three catalytic motifs and (ii) those with divergent ATP-binding and metal-binding motifs. This review aims to provide a comprehensive overview of these recently classified bacterial pseudokinase families, while also exploring the future of bacterial pseudokinase research, including potential applications in real-world settings.

## Bacterial pseudokinases exhibiting divergence in all three canonical catalytic motifs

Pseudokinases exhibiting degradation of all three catalytic motifs are relatively common in prokaryotes. As discussed above, pseudokinases (alongside many canonical kinases) perform critical non-catalytic roles, as ably demonstrated by EssB/YukC [2,3,23,59–61]. In the case of pseudokinases lacking ATP binding, no formal selective pressures constrain the catalytic triad to cater to one specific catalytic function. Subsequently, these pseudokinases nearly always lack ATP-binding capabilities [8], with five of the seven annotated bacterial pseudokinase families currently annotated to this class: ActPs1, ActPs2, ActPs3, GLW, and CP1.

## ActPs1 pseudokinases

**Figure 3:**
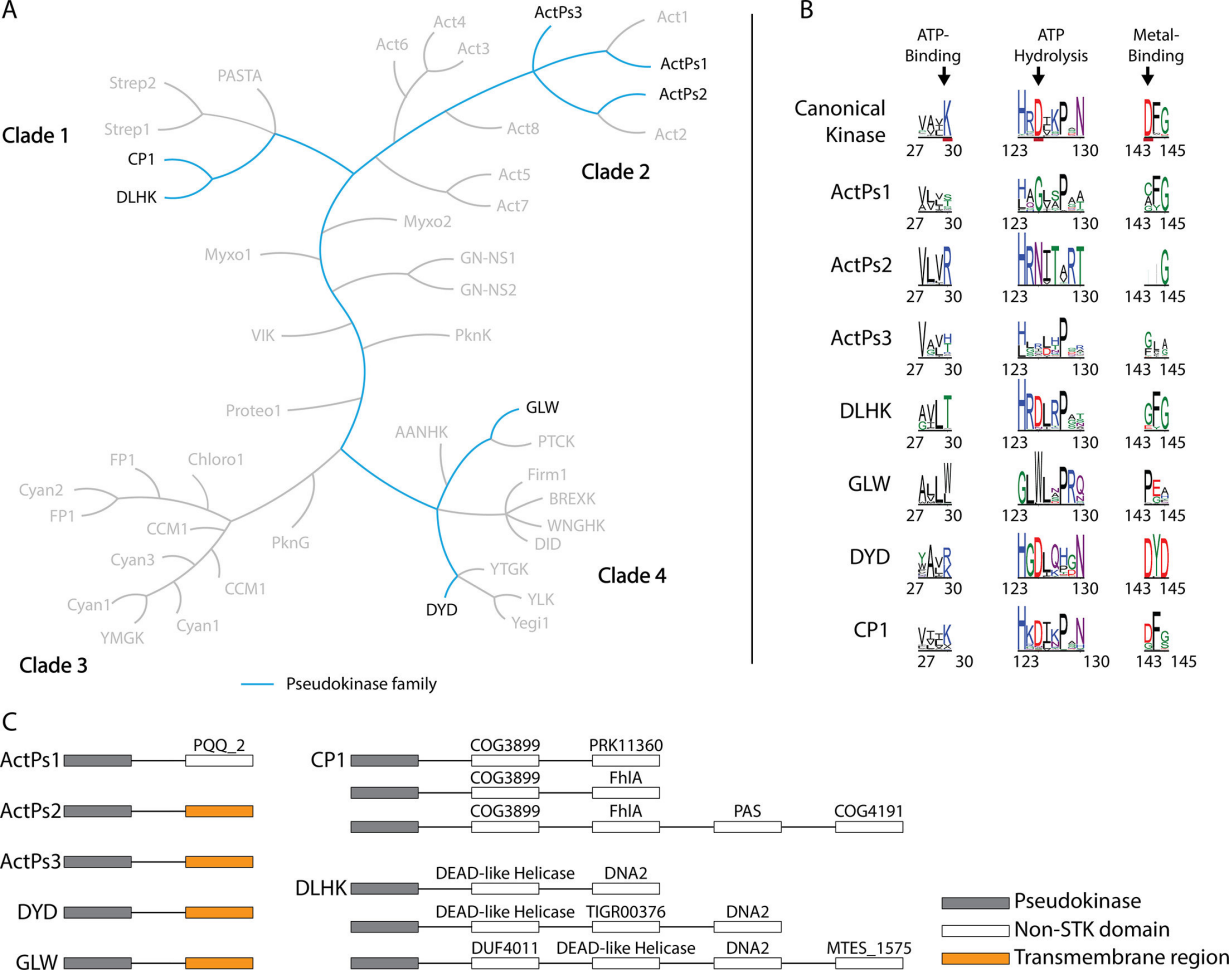
Evolution, catalytic triad sequence logos, and conserved domains of bacterial pseudokinase families. (**A**) The bacterial serine–threonine kinase family tree [72]. The lineage of pseudokinase families is shown in cyan. (**B**) Sequence logos of the motifs of bacterial pseudokinase families. Residues underlined in red are members of the catalytic triad. (**C**) Conserved domains that are associated with each of the bacterial pseudokinase families. Pseudokinase domains are represented with gray blocks, non-STK domains are represented with white blocks, and transmembrane regions are represented with orange blocks. Conserved domain database annotations are shown above the non-STK domains. GLW, glycine-leucine-tryptophan; PAS, Per-Arnt-Sim; PQQ, pyrroloquinoline quinone; STK, serine–threonine protein kinase.

**Figure 4:**
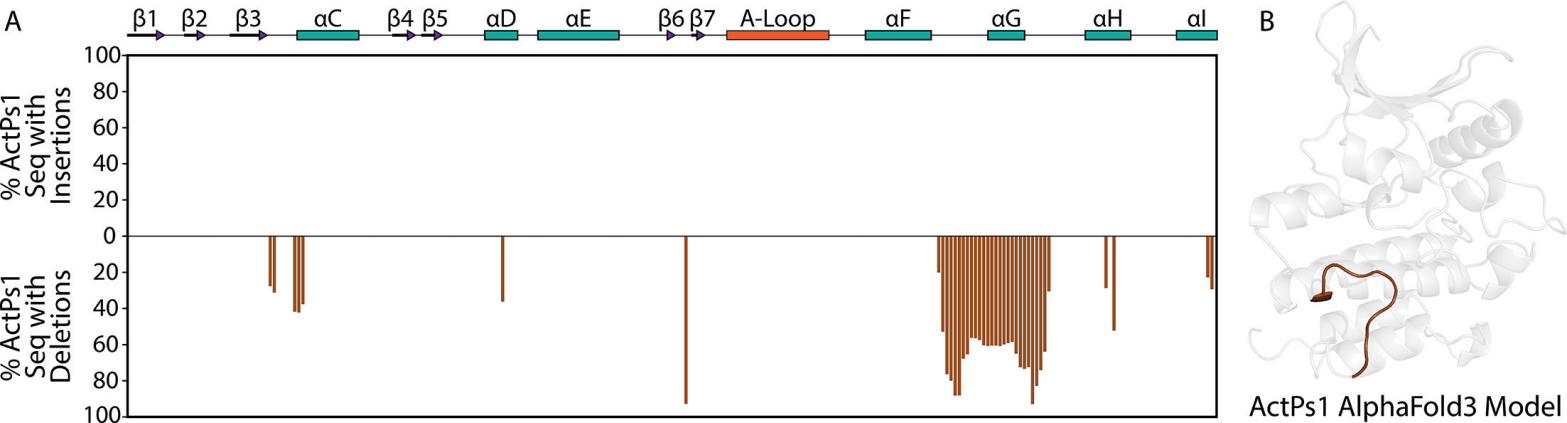
Conserved deletions within ActPs1 sequences.

ActPs1 is a conserved pseudokinase family found primarily in actinobacteria. It closely relates to another bSTK family, Act1 (Figure 3A). ActPs1 kinases have substitutions in all three canonical catalytic residues (Figure 3B), making it unlikely that these kinases can bind to or hydrolyze ATP. An interesting and unique feature of ActPs1 kinases is that about 60% of sequences belonging to this family completely lack the G-helix (Figure 4), a region of the kinase with established roles in protein–protein interactions [76–78]. It is currently unclear how the absence of a G-helix affects the ability of ActPs1 kinases to interact with other proteins. Many ActPs1 kinase-containing polypeptides also possess a pyrroloquinoline quinone (PQQ) 2 domain that lies C-terminal to the pseudokinase domain (Figure 3C). PQQ is a redox-active cofactor that is involved in growth stimulation [79], metabolite oxidation [80–82], ROS response modulation [83–85], and plant–bacteria interactions [86–88]. PQQ domain 2 is one of four domains of PQQ synthase PqqF, which is required for the biosynthesis of PQQ [89]. PQQ has been shown to stimulate the activity of some bSTKs with PQQ-interacting domains [90], suggesting it may play a role in modulating ActPs1 function.

(**A**) Bars below the x-axis (shown in brown) represent the percentage of sequences within the ActPs1 family with a deletion within the alignment. (**B**) A representative AlphaFold3 model of an ActPs1 sequence (NCBI accession number: WP_175290304.1). The region in red indicates the deletion of the G-helix.

## ActPs2 pseudokinases

Like ActPs1, pseudokinases in the ActPs2 family are predominantly found in actinobacteria. Phylogenetic analyses indicate that ActPs2 represents a non-catalytic subfamily within the broader Act2 bSTK family (Figure 3A). ActPs2 kinases exhibit several key catalytic impairments: almost all have a conserved arginine replacing the β3 lysine, the catalytic aspartate in the HRD motif is substituted with an asparagine, and most show a deletion in the metal-binding DFG motif (Figure 3B).

**Figure 5:**
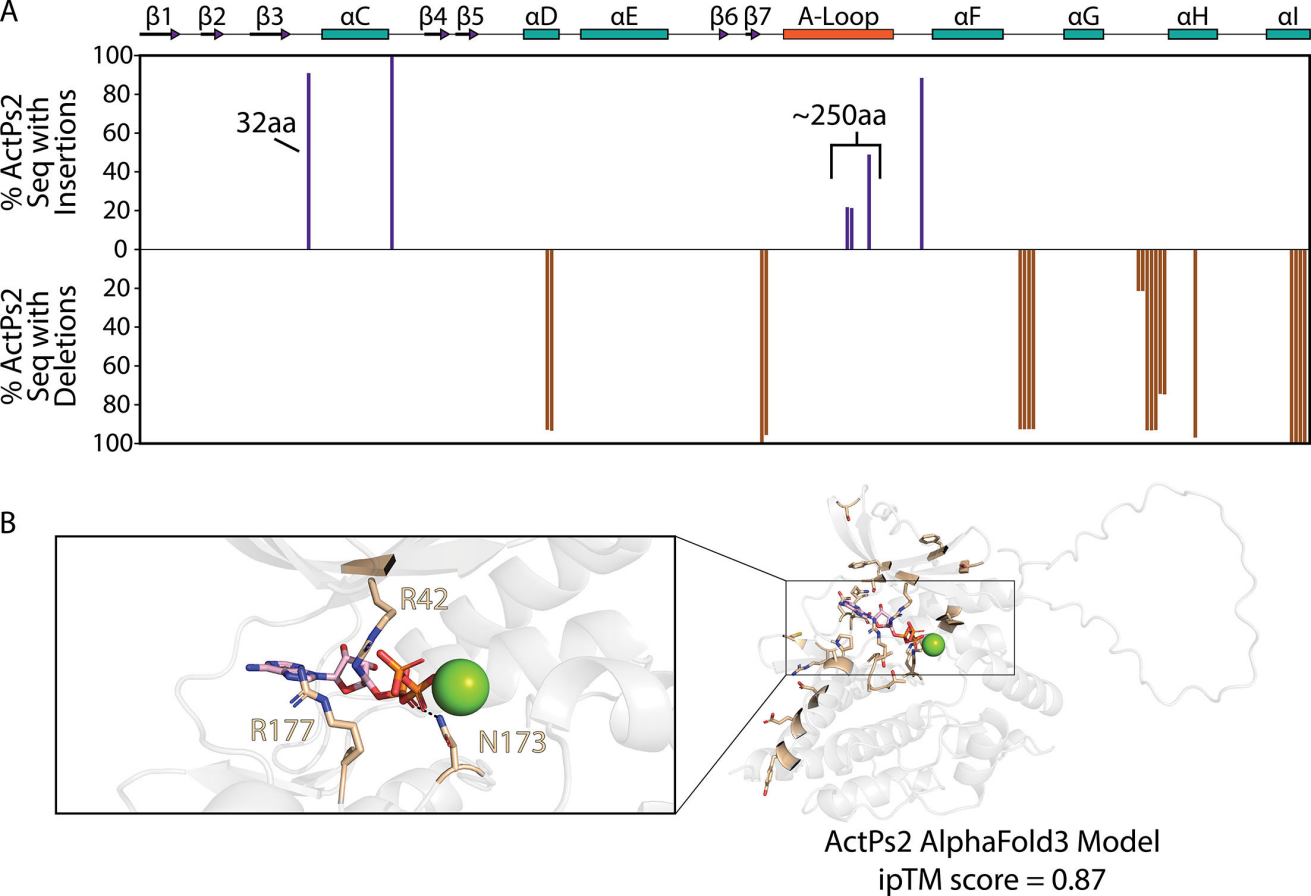
ActPs2 conserved deletions and constraints. (**A**) Conserved insertions and deletions within ActPs2 sequences. Bars above the x-axis (shown in purple) represent the percentage of sequences within the ActPs2 family with an insertion within the alignment. Bars below the x-axis (shown in brown) represent the percentage of sequences within the ActPs2 family with a deletion within the alignment. (**B**) ActPs2 constraint residues within the active site. AlphaFold3 model of an ActPs2 representative (NCBI accession number: WP_053198479.1) bound to ATP and one magnesium ion. Additional (co-evolving) amino acid constraints are shown in light brown.

The conserved β3 lysine-to-arginine substitution in ActPs2 suggests that this bacterial kinase family may have evolved alternative mechanisms for ligand (nucleotide) binding. This phenomenon has been observed in eukaryotic pseudokinases, such as EphB6 [8] and KSR1 [14,91], which retain the ability to bind ATP or non-canonical small molecules despite the β3 lysine-to-arginine mutation, and in catalytically active WNK kinases, in which the β3 lysine is substituted for Cys, with ATP binding promoted by a lysine that has migrated to the β2 strand [92]. AlphaFold3 modeling of a representative ActPs2 kinase predicts ATP-binding capabilities with an iPTM score of 0.87 bound to a single magnesium ion (Figure 5A). Notably, two arginines in the active site, R42 and R177, co-ordinate with the phosphate group of ATP and can potentially contribute to ATP binding. However, further biochemical experimentation is needed to evaluate this hypothesis.

Interestingly, ~90% of ActPs2 kinases contain two notable insertions: a 32-amino-acid segment between the β3 strand and the αC helix, and a remarkable 250-amino-acid extension within the activation loop (Figure 5B). Structural models indicate that both insertions are disordered, suggesting they do not form additional structured domains, leaving their functional significance unclear. Additionally, transmembrane regions are commonly found C-terminal to ActPs2 kinases (Figure 3C), further supporting their membrane-associated functions.

## ActPs3 pseudokinases

**Figure 6:**
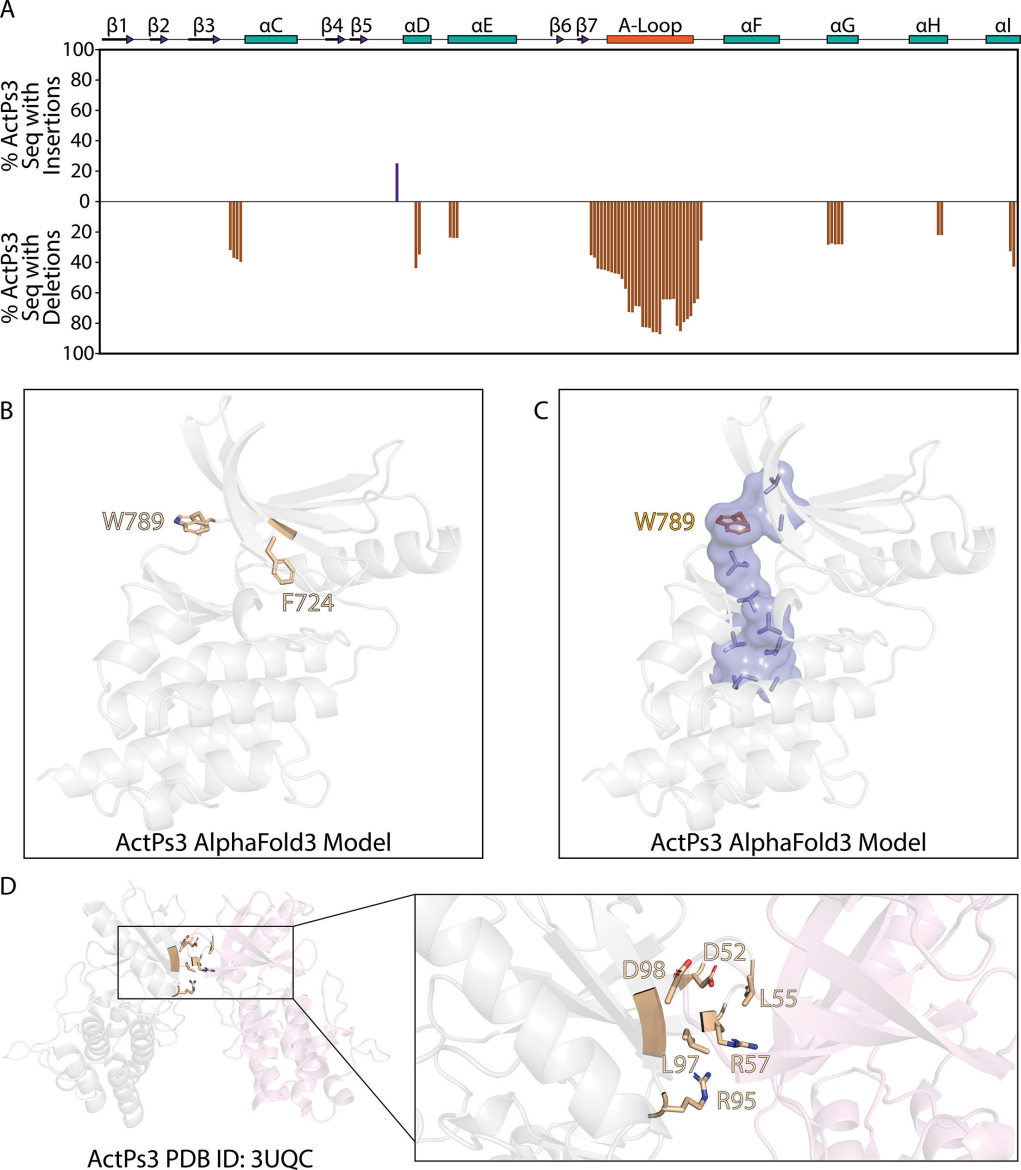
ActPs3 conserved deletions and constraints. (**A**) Conserved insertions and deletions within ActPs3 sequences. Bars above the x-axis (shown in purple) represent the percentage of sequences within the ActPs3 family with an insertion within the alignment. Bars below the x-axis (shown in brown) represent the percentage of sequences within the ActPs3 family with a deletion within the alignment. (**B**) ActPs3 family-specific constraints located within the active site. (**C**) W789 completing the C-spine, shaded in blue, in the MviN pseudokinase domain. (**D**) dimer subunits (shown in gray and pink) and dimer interface between MviN pseudokinase domains (PDB ID: 3UQC). ActPs3 family-specific constraints located at the dimer interface are shown in light brown.

ActPs3 pseudokinases have primarily been annotated in actinobacteria, where they exhibit complete degradation of the canonical kinase catalytic motifs (Figure 3B). In addition, they contain a hallmark deletion in the activation loop, along with several smaller deletions throughout the kinase domain (Figure 6A). Many pseudokinases in this family also feature a transmembrane region located C-terminal to the kinase domain, suggesting a membrane-localized function (Figure 3C).

A notable member of the ActPs3 family is found in *Mycobacterium tuberculosis* (Table 1). This pseudokinase domain is located within MviN, a transmembrane protein essential for peptidoglycan synthesis [24]. Thermal stability analysis has demonstrated that the purified MviN pseudokinase domain is deficient in both ATP and magnesium ion binding [8]. This inability to bind ATP is likely due to constrained bulky aromatic residues that project into the active site [8,24] (F724 and W789 in Figure 6B), physically occupying the ATP-binding pocket to complete a catalytic spine architecture that is associated with the ‘active’ conformation of canonical protein kinases (Figure 6C) [77,93]. However, the C-terminal tail of the MviN pseudokinase domain is phosphorylated by PknB, which facilitates recruitment of the forkhead-associated domain of the FhaA protein. This interaction ultimately inhibits peptidoglycan synthesis. While the MviN pseudokinase domain is not required for peptidoglycan synthesis, it serves as a homeostatic regulator of cell wall metabolism by promoting the recruitment of an inhibitory protein. Although other ActPs3 kinases lack the carbohydrate-binding domain found in *M. tuberculosis* MviN*,* they may also possess scaffolding functions. Within the *M. tuberculosis* genome, the MviN pseudokinase gene is found within an operon containing three other genes, including an ADP-ribose pyrophosphatase, a DNA-directed RNA polymerase subunit, and a third unannotated gene. Additionally, an ActPs3 pseudokinase has been identified in two other pathogens, *Mycobacterium leprae* and *Nocardia asteroides* (Table 1), although their precise function remains unclear.

Interestingly, the MviN pseudokinase domain has been shown to form a back-to-back dimer in solution [24] (Figure 6D), resembling the dimer architecture of some canonical bSTKs. Many ActPs3 family-specific constraints are concentrated in the N-lobe, particularly at the dimer interface. Notably, residues L750 and D793 are crucial for the contact between each pseudokinase subunit and are analogous to those required for dimerization in other bSTKs [94]. Thus, the ActPs3 family-specific constraints are imposed on residues involved in the dimerization of the pseudokinase domain that is predicted to be crucial for signaling output(s).

### GLW pseudokinases

The GLW family of pseudokinases is predominantly found in proteobacteria and derives its name from a distinctive GLW motif, which replaces the catalytic HRD motif of canonical protein kinases (Figure 3B). Another unique feature is the presence of a conserved leucine/tryptophan residue in a poly-leucine motif (LLLL/W) within the active site, replacing the ATP-binding lysine in the β3 strand. The presence of these bulky, hydrophobic residues likely prevents ATP binding. Like many other kinase families, GLW kinases are membrane-associated, with a transmembrane region C-terminal to the kinase domain (Figure 3C).

## CP1 pseudokinases

CP1 pseudokinases are primarily annotated in cyanobacteria and proteobacteria and are closely related to DLHK kinases. Interestingly, ~40% of CP1 kinases are classified as pseudokinases due to variations in one or more catalytic motifs (Figure 3B). Some CP1 kinases possess a single degraded catalytic motif, and others exhibit degradation in a combination of one or more catalytic motifs. Consequently, some CP1 pseudokinases may retain the ability to bind ATP and/or magnesium, while others likely do not. Unlike many other bSTKs, CP1 kinases lack transmembrane regions, indicating cytosolic distribution that could be influenced by binding partners. All CP1 pseudokinases are physically linked to a COG3899 domain—itself a predicted ATPase [95]—and also frequently contain additional domains such as the formate hydrogen lyase transcriptional activator (FhlA) or a Per-Arnt-Sim (PAS) domain (Figure 3C). In *E. coli*, FhlA interacts with formate to initiate transcription of formate hydrogenase [96], a key enzyme in anaerobic energy generation [97]. GAF domains, which are found in FhlAs, and PAS domains are common sensor domains for two-component systems [98,99], a common signaling system in bacteria. The diversity of canonical motif degradation and associated domains suggests a wide range of functions for CP1 pseudokinase-based signaling. Importantly, two CP1 pseudokinases have been identified in the *Leptospira interrogans* serovar Lai (Table 1), a pathogen responsible for leptospirosis and its more severe, sometimes fatal form, Weil’s disease [100]. Leptospirosis is a globally prevalent zoonotic disease transmitted through water and soil contaminated by infected animal urine. Currently, almost nothing is known about the role of CP1 pseudokinases in *L. interrogans*. An operon analysis revealed that one of the pseudokinase genes is the only gene in its operon, and the other is found in an operon with two other unannotated genes.

## Bacterial pseudokinases with divergent ATP- and metal-binding motifs

A subset of divergent bacterial pseudokinase families, including DYD and DLHK kinases, retain canonical HRD and/or DFG motifs. Below, we discuss family-specific constraints that define these pseudokinases and which enable compensatory alternative modes of ATP binding, as noted previously for some of the eukaryotic pseudokinases such as ULK4 and WNK1 [8,92,101].

## DYD pseudokinases

**Figure 7:**
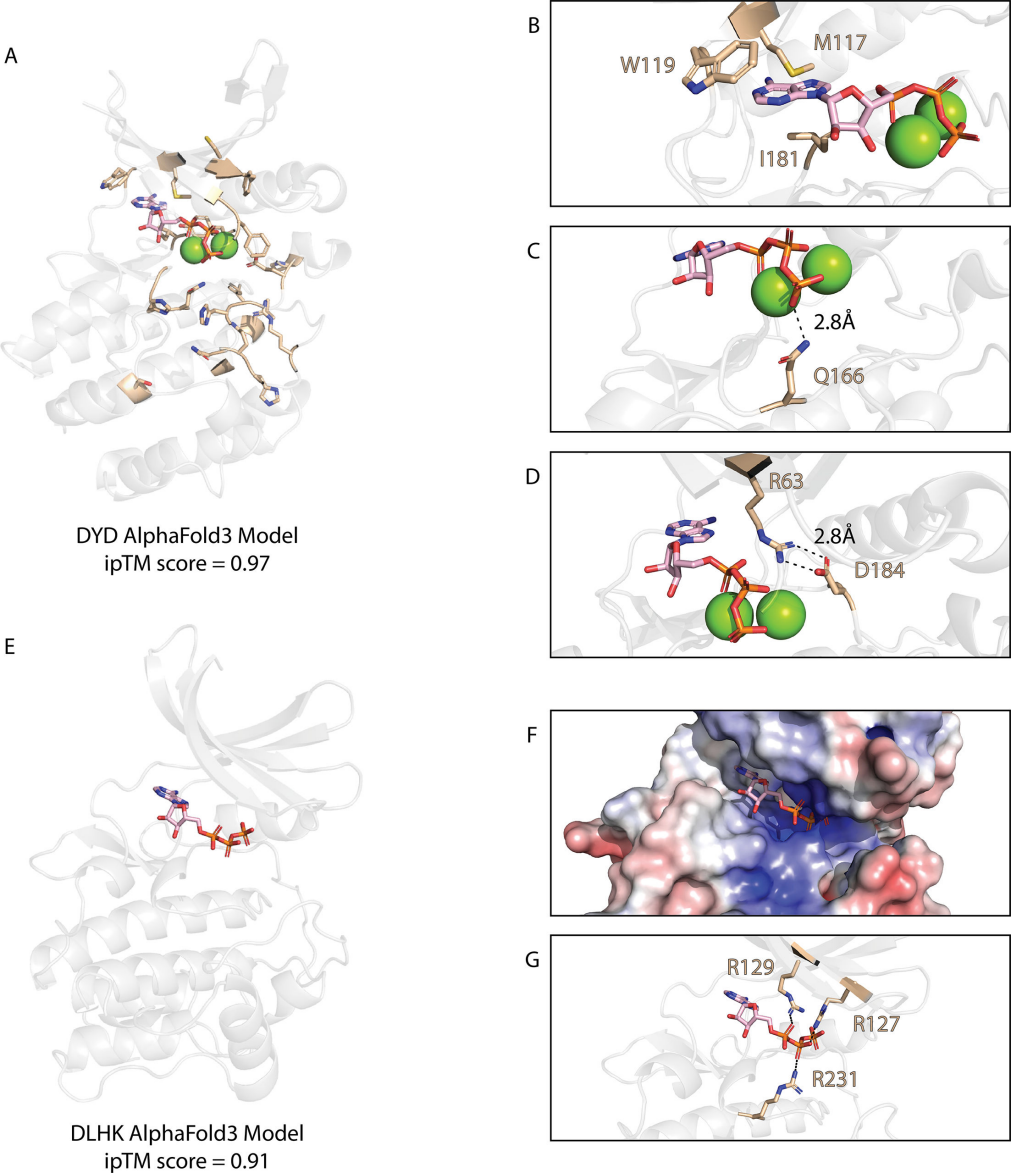
AlphaFold3 models of DYD and DLHK representatives bound to ATP. (**A**) An AlphaFold3 model of a DYD kinase (NCBI accession number: WP_153409036.1) modeled with ATP and magnesium. DYD family-specific constraints are shown in light brown. (**B**) DYD family-specific constraints in the ATP-binding pocket. (**C**) DYD family-specific constraint forming a hydrogen bond with the gamma phosphate of ATP. (**D**) AlphaFold3 model of a DYD kinase (NCBI accession number: WP_153409036.1) bound to ATP and magnesium. A salt bridge is formed between an arginine in the β3 strand and an aspartate of the DYD motif in the activation loop (shown in light brown). (**E**) An AlphaFold3 model of a DLHK kinase (NCBI accession number: WP_188258456.1) modeled with ATP and magnesium. (**F**) Electrostatic distribution within the DLHK AlphaFold3 model. Blue regions indicate a more positive charge, and red regions indicate a more negative charge. (**G**) Three highly conserved arginines, shown in light brown, point into the nucleotide-binding site in the DLHK model and interact with negatively charged nucleotide phosphates. DYD, aspartate–tyrosine–aspartate.

DYD kinases are membrane-associated kinases found in actinobacteria and firmicutes (Figure 3C), and ~60% are annotated as pseudokinases. These pseudokinases are characterized by the substitution of the ATP-binding lysine with an arginine (Figure 3B) alongside the absence of the conserved αC-helix glutamate, which is involved in stabilizing the active conformation of most kinases, including atypical kinases such as WNK1. Despite these structural deficiencies, AlphaFold3 models ATP in the pocket with high confidence (iPTM score of 0.97) in DYD kinases (Figure 7A). Although this prediction has to be experimentally evaluated, several factors suggest ATP binding in DYD kinases. Firstly, three hydrophobic DYD family-specific constraints in the ATP-binding site form a hydrophobic pocket, stabilizing the adenosine of the ATP (Figure 7B). The second ATP stabilizing feature is an asparagine within the catalytic loop (Figure 7C), which forms a hydrogen bond with the gamma phosphate of the ATP. Finally, the second aspartic acid of the unique DYD motif (from which the family derives its name) forms a salt bridge with the β3 arginine of the ATP-binding motif (Figure 7D), which may compensate for the missing C-helix glutamate whose ionic interaction with the canonical β3 lysine defines the ‘C-helix-in’ conformation associated with catalytic activity. A similar mechanism is observed in protein *O*-mannose kinase (POMK), a eukaryotic protein kinase that phosphorylates O-mannose rather than proteins [102,103]. Despite lacking the canonical β3 lysine and αC-helix glutamate, POMK, formerly protein kinase-like protein SGK196 retains ATP-binding capabilities by forming a salt bridge between a conserved lysine in the G-loop and a glutamate in a conserved DLD motif, which replaces the typical DFG motif [104].

## DLHK pseudokinases

DLHK pseudokinases are primarily found in proteobacteria and share a close evolutionary relationship with the CP1 pseudokinase family. DLHK kinases are often linked to diverse (non-kinase) protein domains, the most common being DEAD-like helicase and DNA2 (Figure 3C). DEAD-like helicases have been shown to function in *E. coli* as ATP-dependent RNA or DNA helicases involved in RNA degradation [105], while DNA2 domains exhibit ATP-dependent nuclease and helicase activity [106]. Within the *Pseudomonas aeruginosa* genome, the DLHK pseudokinase gene is found in an operon with three other genes: an undefined ATPase, an organic radical activating enzyme, and a third unannotated gene. Unlike many other bSTK families, DLHK family members all lack transmembrane regions, suggesting they are not integrally membrane-associated.

Notably, DLHK proteins possess the catalytic aspartate of the HRD motif, suggesting possible retention of catalytic activity (although to our knowledge, this has not been experimentally validated) despite notable degradation of ATP- and DFG metal-binding motifs. They also contain a conserved asparagine residue situated at the end of the ‘HRD’ loop, which participates in metal binding in canonical kinases (Figure 3A). Remarkably, AlphaFold3 models of the representative DLHK kinase complexed with ATP suggest an alternative mode of ATP binding (Figure 7E). The electrostatics reveal that the active site of the kinase domain is highly positively charged (Figure 7F), due to the presence of three arginine side chains that extend into the active site (Figure 7G). One of these arginines, R231, is a DLHK family-specific constraint within the catalytic loop. The other two arginines are not constrained but are highly conserved within the β2 strand of DLHK kinases [72]. These features are reminiscent of the ATP-binding mechanism observed in ULK4, which also binds ATP in a metal-independent manner due to multiple highly conserved positively charged residues within the active site [101,107,108]. Although experimental validation is required, these structural features may suggest that members of the DLHK family can bind ATP despite lacking the canonical β3 lysine.

## Discussion

Multidrug-resistant bacterial infections are ranked among the leading causes of global mortality and are projected to have caused 40 million deaths by 2050, rising to ~2 million deaths per annum worldwide [109]. Most clinically approved antibiotics are derivatives of small molecules that disrupt a narrow range of microbial biosynthetic pathways, including the well-known β-lactam class. Compounds exhibiting alternative modes of action remain scarce in the clinic [110], exacerbating the spread of MDR bacteria. However, bSTKs and their pseudokinase counterparts are highly homologous structurally to eukaryotic S/T/Y kinases (Figure 1), for which ~180 FDA-approved clinical compounds exist, nearly all for indications in oncology and inflammatory disease spaces. It is therefore intriguing that bSTKs, including the vast number of pseudokinases that form part of the bSTK catalogue, represent an ‘undrugged’ pool of new potential therapeutic targets [111–116]. One example where this approach has been tested is for prokaryotic aminoglycoside kinases, which inactivate antibiotics such as kanamycin through phosphorylation and induce drug resistance. These are intriguing ‘off-targets’ for pre-existing ATP-site small molecules designed to target ePKs, and combined target-hopping and optimization within the bSTKs might be an innovative approach to develop antibiotics of the future [117,118]. Importantly, several pseudokinases discussed in this review originate from pathogenic or opportunistically pathogenic bacteria. For example, YukC and its ortholog, EssB, expressed by *B. subtilis* and *S. aureus*, respectively, are associated with hospital-acquired infections [119–121], and the pseudokinase domains are crucial for maintaining appropriate virulence-determining T7SSb function [23,57–61]. Given the number of pseudokinase families predicted to be membrane-associated (Figure 3C), similar roles in bacterial effector secretion are possible, even more likely. Moreover, some bacterial pseudokinases may also directly function as survival-promoting effector proteins, such as the polyglutamase SidJ [66,67]. Understanding the function and activities of bacterial pseudokinases is therefore crucial to elucidate their roles in infection and virulence and to explore their utility as targets for antimicrobial compounds.

In 2022, the FDA approved the first pseudokinase-targeting drug, Deucravacitinib/Sotyktu [122], to treat moderate-to-severe plaque psoriasis. Remarkably, Deucravacitinib allosterically regulates the catalytic output of the tyrosine kinase 2 (TYK2) by binding directly to the pseudokinase domain and locking it in an inactive state. Because they often lack classical catalytic activity, pseudokinases are not constrained to a single function, which enables evolutionary adaptation of the kinase fold to serve a wide range of roles. Moreover, pseudokinases often have unique, non-canonical structural and regulatory features, which may include novel druggable pockets containing selectivity determinants for inhibitor compounds, conceptually minimizing off-target effects when compared with their canonical counterparts [123–126]. Such similarities have stymied the development of isoform-specific inhibitors of the JAK/TYK canonical kinase domains, an issue that can be effectively addressed through drugging the less-constrained sequences found in the pseudokinase domains of individual JAK/TYK polypeptides [122]. As research continues to uncover their functions, we propose that bacterial pseudokinases will also emerge as novel targets for precision antibiotics, expanding the arsenal of strategies to combat infectious diseases and antimicrobial resistance.

In order to achieve this, it should be recognized that research on bSTKs in general, notably pseudokinase-defined sequences within these families, remains very limited, and several challenges must be addressed as the field expands. One of the most significant hurdles is the experimental functional characterization of each of these proteins. While human and other eukaryotic proteomes receive extensive attention due to their direct relevance to human health, the availability of comprehensive bacterial proteomes is limited to just a handful of organisms with clear medical, agricultural, or industrial importance [127]. Even among well-studied species, bacterial pseudokinase research has been neglected. For example, the genome of *P. aeruginosa,* a common opportunistic pathogen responsible for severe hospital-acquired infections, such as pneumonia and sepsis [128], and one of the most important pathogens in cystic fibrosis patients [129] encodes an unstudied DLHK pseudokinase (Table 1). *P. aeruginosa* is currently classified as a significant health threat by the World Health Organization and Centers for Disease Control due to its multidrug resistance [130–133]. Despite this, the DLHK pseudokinase of *P. aeruginosa* has evaded functional characterization, limiting our understanding of bacterial pseudokinase functions. A critical first step would be to characterize a representative pseudokinase from each family discussed in this study. In this regard, the unique family-specific constraints delineated in this study, combined with experimental evaluation of ATP-binding properties using thermal shift assays developed for eukaryotic pseudokinases [8], can provide a foundation for broader functional insights and expand our knowledge of bacterial pseudokinase roles.

A second major challenge is the sheer volume of genetic and proteomic data now available. The rise of multi-omics studies has led to an explosion of prokaryotic sequence information [134,135], far outpacing the capacity of traditional bioinformatics approaches to analyze it efficiently. This data deluge will take many years to process manually [136,137], but recent advances in machine learning offer a potential solution. By integrating ‘artificial intelligence,’ particularly recent advances in evolutionary scale models, researchers can more rapidly identify pseudokinases within bacterial genomes [138], determine their genomic context (including operon structure [139]), and predict their involvement in cellular metabolic pathways [136,140,141]. Such approaches could accelerate discoveries and aid in the identification and characterization of divergent bacterial pseudokinases such as SidJ, YukC, and SelO [27,142], which are, by definition, difficult to detect using traditional bioinformatics approaches but can be readily assayed, both biochemically and *in vivo*.

## Conclusions

The past 15 years have been crucial for establishing a baseline knowledge from which more-detailed bacterial pseudokinase analysis is slowly emerging. Recent studies have shown that bacterial pseudokinases (i) form families of exclusively pseudokinase bSTKs, (ii) are involved in major bacterial pathways including virulence, cellular development, and antibiotic resistance, and (iii) possess unique, conserved structural elements and motifs such as significantly extended activation loops in ActPs2 pseudokinase and different modes of ATP-binding (notably a DYD motif in DYD pseudokinase). These findings provide valuable insights into kinase evolution and bacterial life cycles and identify key motifs and sequence indels for functional characterization. However, an urgent priority remains in understanding their relevance to human health. Employing machine-learning algorithms alongside high-throughput genetic screening can accelerate bSTKs phenotype screens. Moreover, by employing some of the tricks-of-the-trade established for the analysis of human pseudokinases, which culminated in the clinical approval of TYK2 ‘allosteric’ (pseudokinase) modulators, bacterial pseudokinases represent a highly appealing area for biomedical research in the next decade.

**Perspectives**

- Pseudokinases lack key catalytic residues that are required for phosphorylation, generally rendering them catalytically inactive. Although historically understudied, bacterial pseudokinases are now recognized as pivotal regulators of bacterial functions despite lacking canonical protein kinase activity.
- Seven distinct bacterial pseudokinase families were recently identified through comprehensive bioinformatic analysis, revealing an unexpected level of structural and functional diversity and providing strong evidence for their broader relevance and roles in bacterial signaling networks.
- Future functional studies focused on these newly identified pseudokinase families will be essential to elucidate their specific biological roles in bacterial physiology and pathogenesis and may ultimately uncover novel targets for the development of antibacterial therapies.

## Open Access

This article has been published open access under our Subscribe to Open programme, made possible through the support of our subscribing institutions, learn more here: https://portlandpress.com/pages/open_access_options_and_prices#conditional

## Abbreviations

AMP: adenine monophosphate
DYD: aspartate–tyrosine–aspartate
FhlA: formate hydrogen lyase transcriptional activator
GLW: glycine-leucine-tryptophan
PAS: Per-Arnt-Sim
POMK: protein O-mannose kinase
PQQ: pyrroloquinoline quinone
PTMs: post-translational modifications
S/T: serine/threonine
STKs: serine–threonine protein kinases
TYK2: tyrosine kinase 2
bSTK: bacterial STK
